# Supplementary material for: A toolkit for mapping cell identities in relation to neighbors reveals conserved patterning of neuromesodermal progenitor populations
Source: PLoS Biol. 2025 Jul 15;23(7):e3003244. doi: 10.1371/journal.pbio.3003244 (PMC12303391; doi:10.1371/journal.pbio.3003244)
Supplement: S1 Fig — Example of the algorithm modules on a segmented six-somite pair embryo with the epiblast manually labeled. Points represent nuclei centroids and XYZ refer to coordinates in the raw image planes. In this process (a) first, the position of each nuclei in the anterior-posterior axis calculated relative to the nearest point on a principal curve in the Y and Z planes, which roughly correspond to the anterior/posterior (A/P) and dorsal/ventral axes, respectively. The dorsal/ventral axis is initially estimated as the distance to the nearest point on this A/P principal curve. The output of this (b) is carried to the next step (b) to identify the position along the left/right (L/R) and the dorsal/ventral (D/V) axis. In this step, sections of the epiblast parallel to the new A/P axis are isolated, four example sections are shown. In (c) Relative positions of nuclei to a principal curve in the X plane (roughly corresponds to the left/right axis) and the estimated D/V axis [distance to the A/P principal curve from (a) are calculated to identify the L/R and D/V axes. (d) The distances of each nuclei along the principal curve are normalized to the ‘midline’, which is the average L/R position of the 90th percentile of spatially smoothed TBXT values. Data for S1 Fig (A–D): Data file 1, https://doi.org/10.5281/zenodo.15802710. (DOCX) [file pbio.3003244.s001.docx]

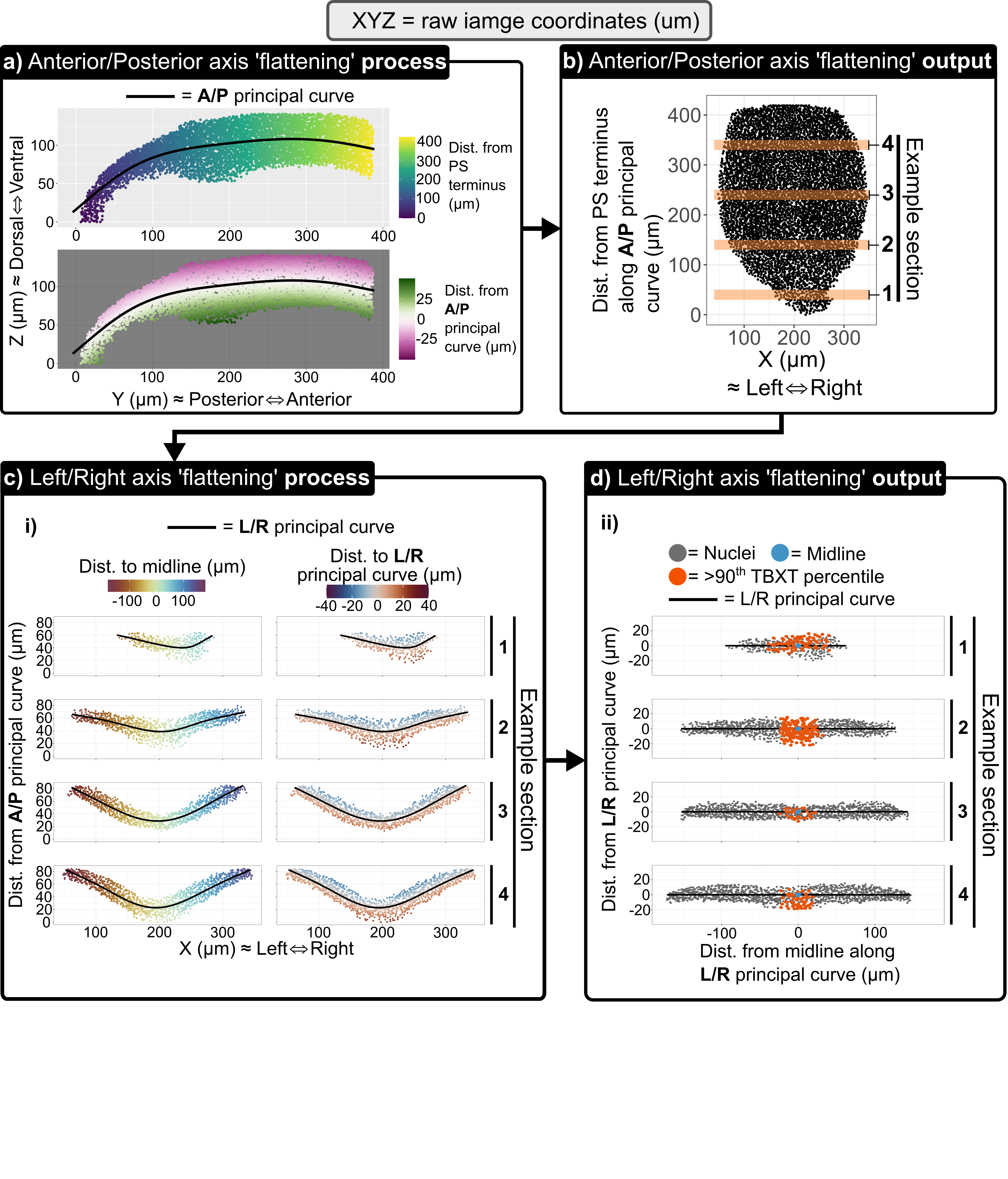
Fig S1 3D Manifold projection and alignment

Example of the algorithm modules on a segmented six somite pair embryo with the epiblast manually labelled. Points represent nuclei centroids and XYZ refer to coordinates in the raw image planes. In this process (**a)** first the position of each nuclei in the anterior posterior axis calculated relative to the nearest point on a principal curve in the Y and Z planes, which roughly correspond to the anterior/posterior (A/P) and dorsal/ventral axes respectively. The dorsal/ventral axis is initially estimated as the distance to the nearest point on this A/P principal curve. The output of this **(b)** is carried to the next step (**b)** to identify the position along the left/right (L/R) and the dorsal/ventral (D/V) axis. In this step, sections of the epiblast parallel to the new A/P axis are isolated, four example sections are shown. In **(c)** Relative positions of nuclei to a principal curve in the X plane (roughly corresponds to the left/right axis) and the estimated D/V axis [distance to the A/P principal curve from **(a)** are calculated to identify the L/R and D/V axes.

**(d)** The distances of each nuclei along the principal curve are normalised to the ‘midline’, which is the average L/R position of the 90^th^ percentile of spatially smoothed TBXT values.

Data for Figure S1(A-D): S1_Data.xlsx https://doi.org/10.5281/zenodo.15531855
