# Supplementary material for: A toolkit for mapping cell identities in relation to neighbors reveals conserved patterning of neuromesodermal progenitor populations
Source: PLoS Biol. 2025 Jul 15;23(7):e3003244. doi: 10.1371/journal.pbio.3003244 (PMC12303391; doi:10.1371/journal.pbio.3003244)
Supplement: S2 Fig — Example of the manual marking of the posteriormost end of the notochord in a confocal stack of four somite pair embryo stained for LaminB1, SOX2, TBXT, and TBX6, with orthogonal views. (DOCX) [file pbio.3003244.s002.docx]

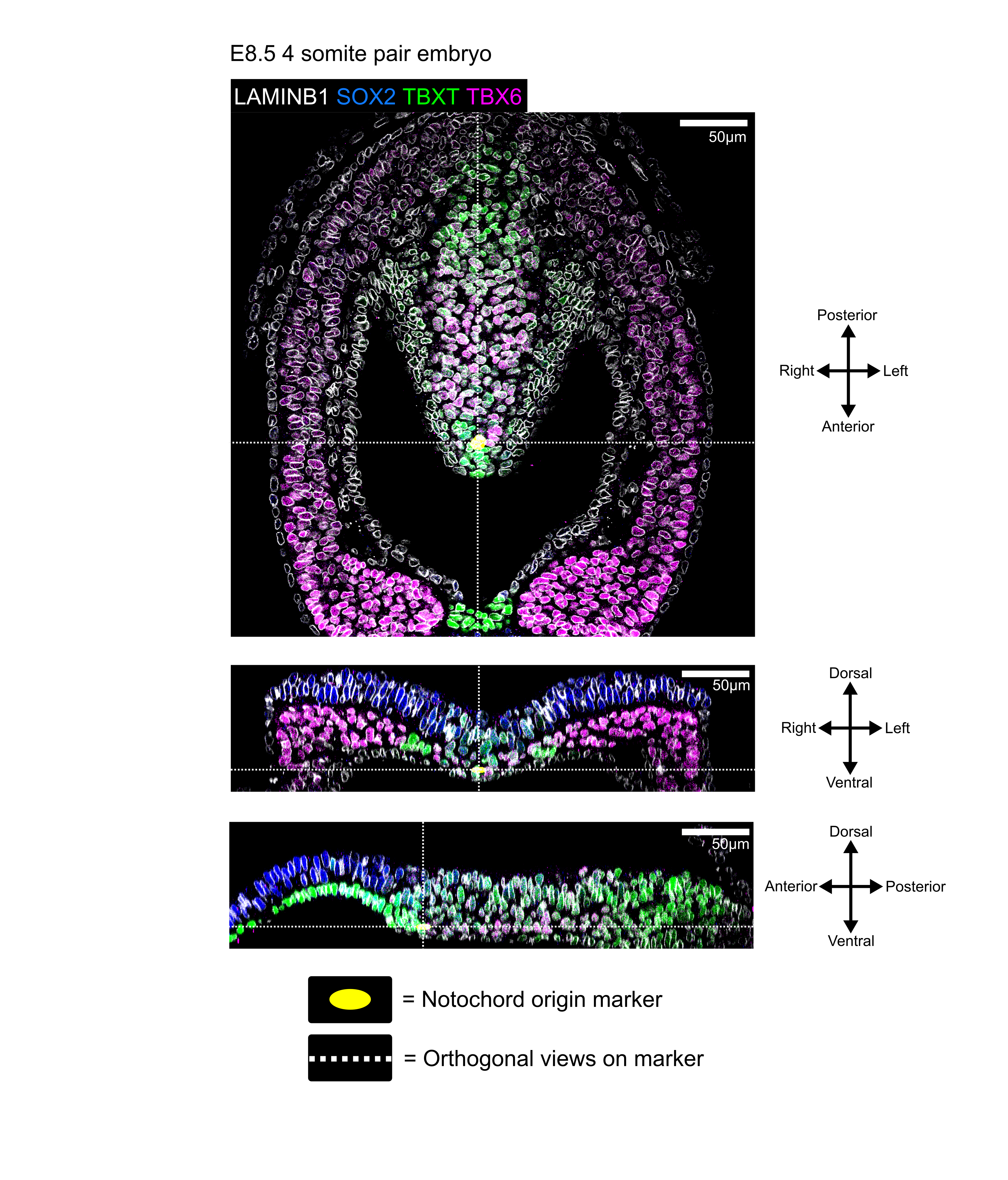


Fig S2 Locating embryo landmarks

Example of the manual marking of the posteriormost end of the notochord in a confocal stack of four somite pair embryo stained for LaminB1, SOX2, TBXT, and TBX6, with orthogonal views.
