# Supplementary material for: A toolkit for mapping cell identities in relation to neighbors reveals conserved patterning of neuromesodermal progenitor populations
Source: PLoS Biol. 2025 Jul 15;23(7):e3003244. doi: 10.1371/journal.pbio.3003244 (PMC12303391; doi:10.1371/journal.pbio.3003244)
Supplement: S3 Fig — Four somite pair embryo epiblast projections showing manual best-fits of pseudospace gates to bifated regions (in boxes). Showing left/right asymmetry in replicate three and non-specific bi-fated region labeling in replicate 5. Data for S3 Fig. Data file 3, https://doi.org/10.5281/zenodo.15802710. (DOCX) [file pbio.3003244.s003.docx]

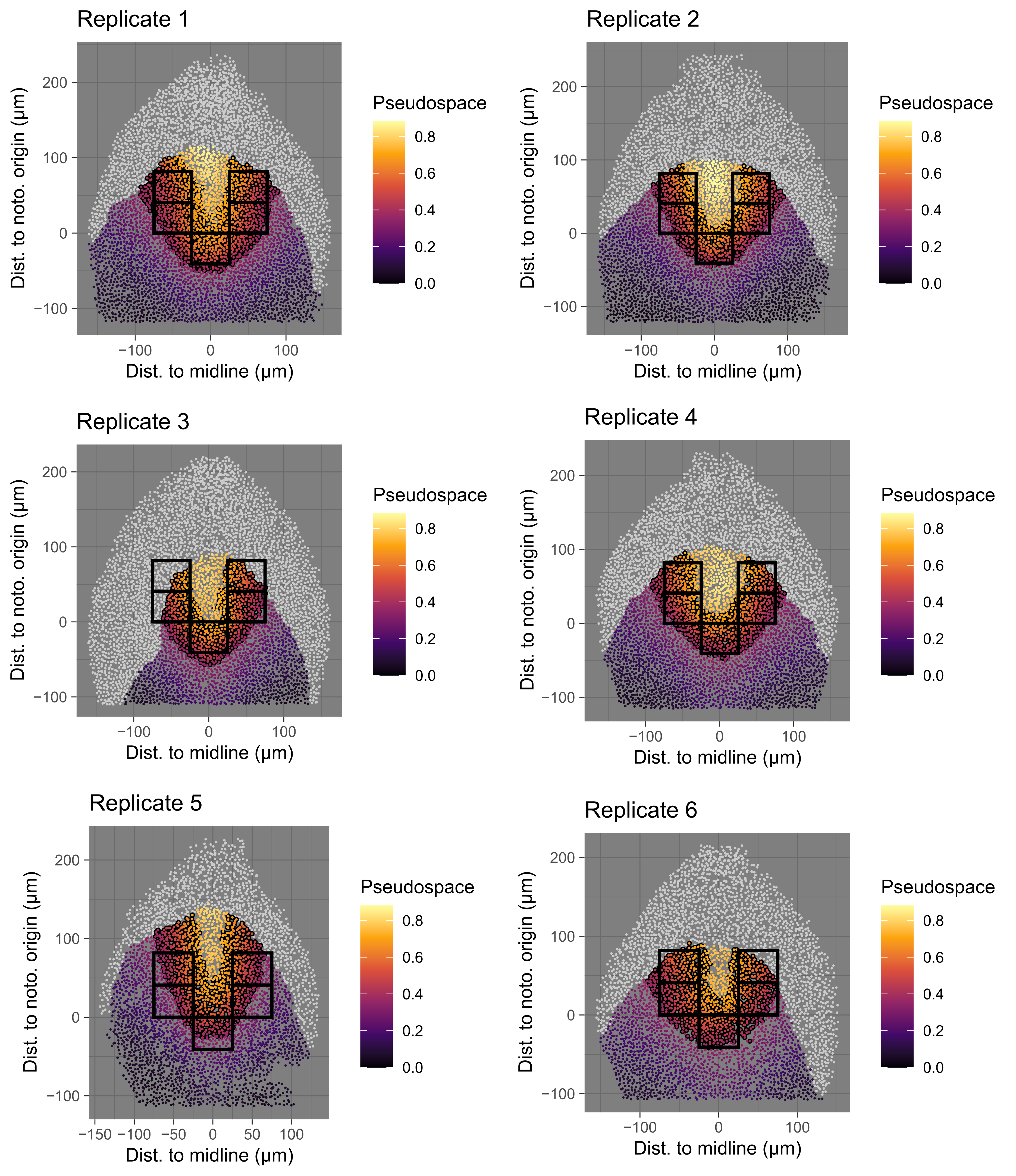


Fig S3 Mapping putative NMP regions

Four somite pair embryo epiblast projections showing manual best-fits of pseudospace gates to bifated regions (in boxes). Showing left/right asymmetry in replicate three and non-specific bi-fated region labelling in replicate 5.

Data for Figure S3 S3_Data.xlsx https://doi.org/10.5281/zenodo.15531855
