# Supplementary material for: A toolkit for mapping cell identities in relation to neighbors reveals conserved patterning of neuromesodermal progenitor populations
Source: PLoS Biol. 2025 Jul 15;23(7):e3003244. doi: 10.1371/journal.pbio.3003244 (PMC12303391; doi:10.1371/journal.pbio.3003244)
Supplement: S4 Fig — An explanation of the method for calculating local gradient direction and steepness. Black arrows indicate positive values while red arrows indicate negative values. (DOCX) [file pbio.3003244.s004.docx]

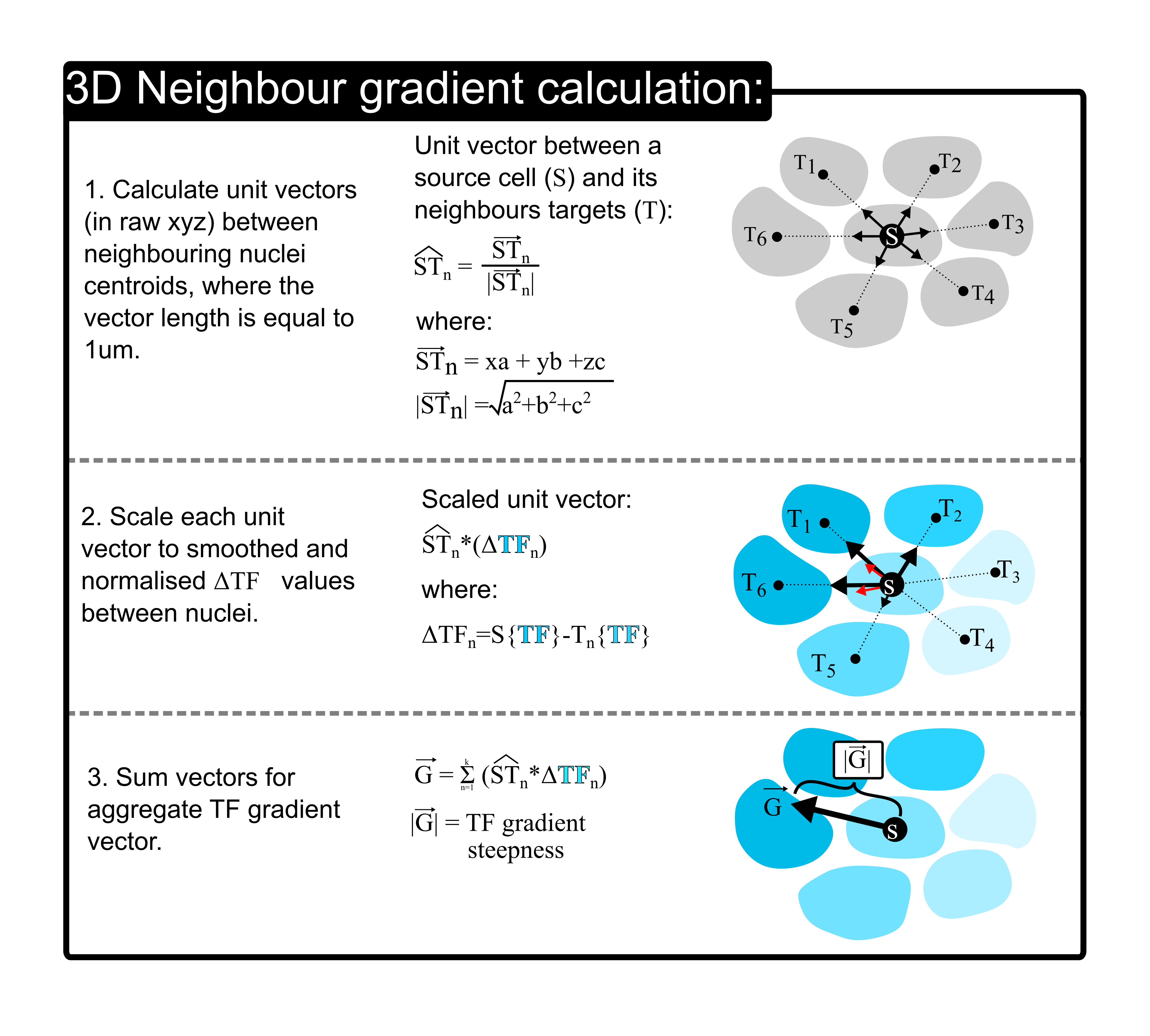


**Fig S4 3D gradient neighbour calculation method**

An explanation of the method for calculating local gradient direction and steepness. Black arrows indicate positive values while red arrows indicate negative values.
