# Supplementary material for: A toolkit for mapping cell identities in relation to neighbors reveals conserved patterning of neuromesodermal progenitor populations
Source: PLoS Biol. 2025 Jul 15;23(7):e3003244. doi: 10.1371/journal.pbio.3003244 (PMC12303391; doi:10.1371/journal.pbio.3003244)
Supplement: S5 Fig — a) Outline of scenarios to explain how log(x/y) is suitable to describe the relationship between two variables. (i) In this scenario, x/y does not equal y/x, and y has logarithmic and asymmetric influence on the output of the calculation. (ii) Using log(x/y) makes the calculation symmetric around 0, but the output is now non-linear. (iii) If the variables x and y can be assumed to be non-linear and a log transformation of x and y is appropriate, then the relationships between log(x) and log(y) with log(x/y) is now linear due to the log law log(x/y) = log(x) − log(y). (b) Summary of the scenarios and dynamic of the calculation output. (DOCX) [file pbio.3003244.s005.docx]

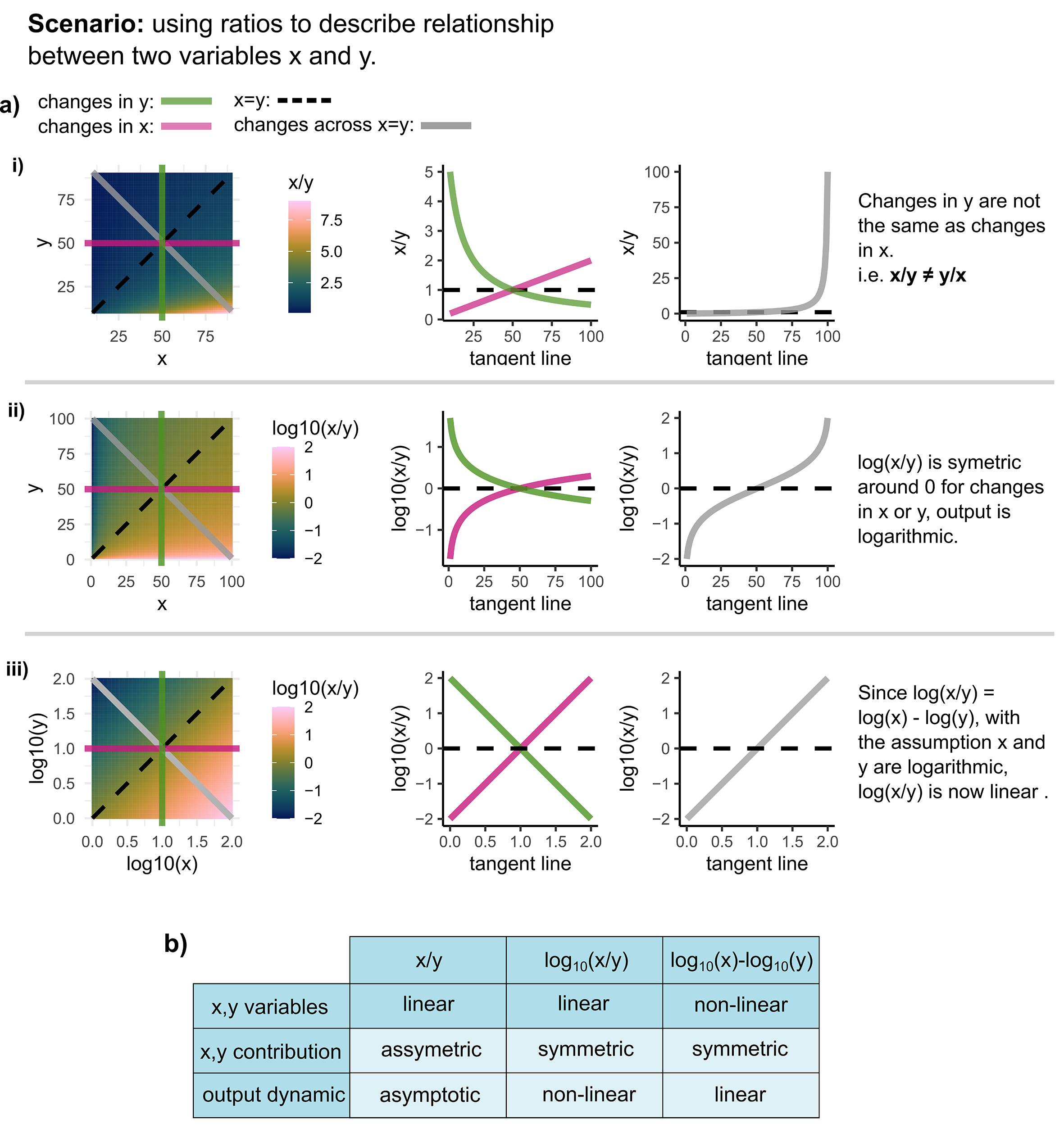


**Fig S5 Using ratios to describe relationship between two variables**

**a)** Outline of scenarios to explain how log(x/y) is suitable to describe the relationship between two variables. **i)** In this scenario, x/y does not equal y/x, and y has logarithmic and asymmetric influence on the output of the calculation. **ii)** using log(x/y) makes the calculation symmetric around 0, but the output is now non-linear. **iii)** If the variables x and y can be assumed to be non-linear and a log transformation of x and y is appropriate, then the relationships between log(x) and log(y) with log(x/y) is now linear due to the log law log(x/y) = log(x) – log(y).

**b)** Summary of the scenarios and dynamic of the calculation output.
