# Supplementary material for: A toolkit for mapping cell identities in relation to neighbors reveals conserved patterning of neuromesodermal progenitor populations
Source: PLoS Biol. 2025 Jul 15;23(7):e3003244. doi: 10.1371/journal.pbio.3003244 (PMC12303391; doi:10.1371/journal.pbio.3003244)
Supplement: S6 Fig — a) Process to produce synthetic data. (b) Comparison of global population distribution for observed and synthetic SOX2, TBXT, and TBX6 signal intensity values. One four somite pair embryo dataset shown. (c) Density plots to compare observed and synthetic TF values of individual nuclei, showing close associations of SOX2 and TBXT, but less accurate TBX6 associations. All embryos shown n = 19. Data for S6 Fig (B–C): Data file 1, https://doi.org/10.5281/zenodo.15802710. (DOCX) [file pbio.3003244.s006.docx]

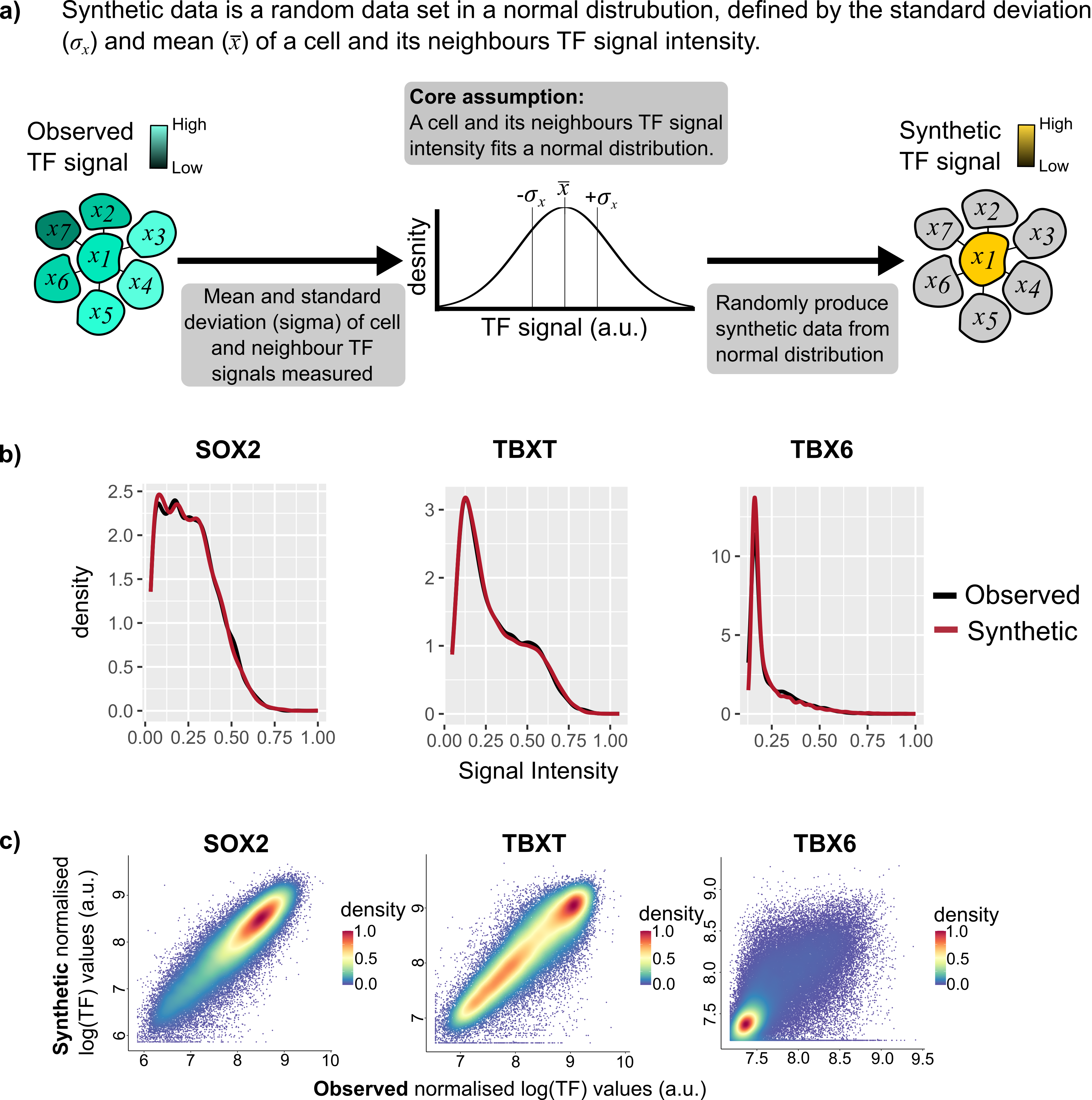


**Fig S6 Synthetic data**

**a)** Process to produce synthetic data.

**b)** Comparison of global population distribution for observed and synthetic SOX2, TBXT, and TBX6 signal intensity values. One four somite pair embryo dataset shown.

**c)** Density plots to compare observed and synthetic TF values of individual nuclei, showing close associations of SOX2 and TBXT, but less accurate TBX6 associations. All embryos shown n=19.

Data for Figure S6 (B-C): S1_Data.xlsx https://doi.org/10.5281/zenodo.15531855
