# Supplementary material for: A toolkit for mapping cell identities in relation to neighbors reveals conserved patterning of neuromesodermal progenitor populations
Source: PLoS Biol. 2025 Jul 15;23(7):e3003244. doi: 10.1371/journal.pbio.3003244 (PMC12303391; doi:10.1371/journal.pbio.3003244)
Supplement: S7 Fig — (A) High throughput single cell quantification method used to complement high resolution imaging (used in spatial neighbor analysis) by boosting sample sizes and N numbers. hNMP monolayers typically form dense structures which are difficult to segment and require time-consuming high-resolution images. To address this, cells are replated as single cells at a lower density and then quickly fixed to perform IF and stain for hNMP markers and DAPI. These are imaged on high imaging content platforms and present a simple challenge for single cell segmentation methods even at low resolutions. (B) Differentiation of human MShef7 to hNMPs with 20 ng bFGF and 2 µM or 3 µM CHIR (as described in Fig 6A) both produce high proportions of TBXT + SOX2 + populations with no statistically significant difference between the groups. (n = 5). (C) CHIR titration of the hNMP differentiation protocol shows the influence of CHIR on SOX2, TBXT, and TBX6, where 3 μM CHIR produces a population with higher TBXT/TBX6 and lower SOX2 than 2 μM (n = 9). Points on violin superplot and barplot show mean per replicate. All error bars indicate confidence intervals of 0.95. Statistical tests performed by one way ANOVA, ** = p < 0.01, *** = p < 0.001. Data for S7 Fig (B–C): Data file 2, https://doi.org/10.5281/zenodo.15802710. (DOCX) [file pbio.3003244.s007.docx]

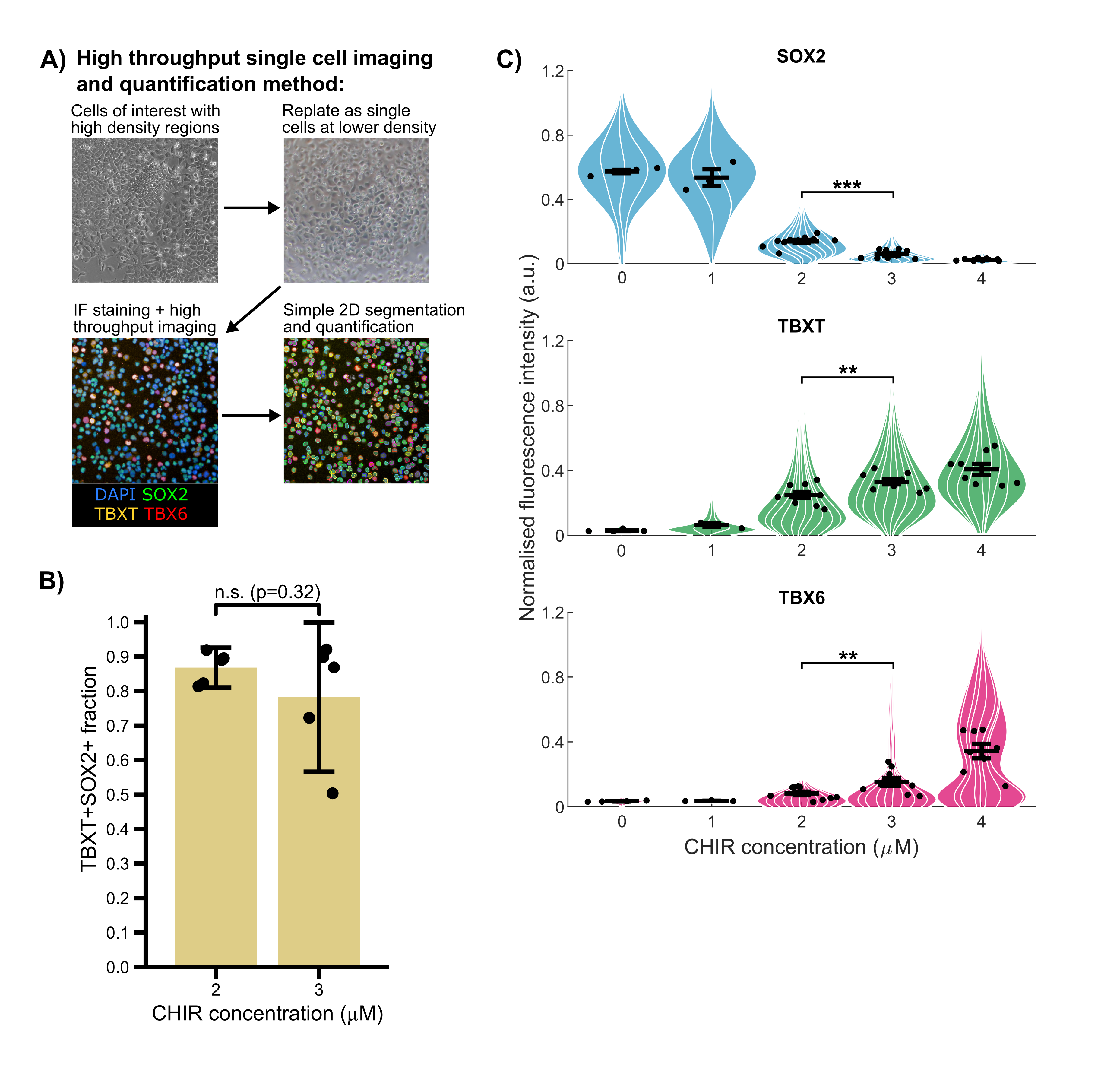


**Fig S7 Human ESC to NMP differentiation characterisation and efficiency.**

**A)** High throughput single cell quantification method used to complement high resolution imaging (used in spatial neighbour analysis) by boosting sample sizes and N numbers. hNMP monolayers typically form dense structures which are difficult to segment and require time-consuming high-resolution images. To address this, cells are replated as single cells at a lower density and then quickly fixed to perform IF and stain for hNMP markers and DAPI. These are imaged on high imaging content platforms and present a simple challenge for single cell segmentation methods even at low resolutions. **B)** Differentiation of human MShef7 to hNMPs with 20ng bFGF and 2uM or 3uM CHIR (as described in figure 6a) both produce high proportions of TBXT+SOX2+ populations with no statistically significant difference between the groups. (n=5). **C)** CHIR titration of the hNMP differentiation protocol shows the influence of CHIR on SOX2, TBXT, and TBX6, where 3μM CHIR produces a population with higher TBXT/TBX6 and lower SOX2 than 2μM (n=9). Points on violin superplot and barplot show mean per replicate. All error bars indicate confidence intervals of 0.95. Statistical tests performed by one way ANOVA, ** = p<0.01, *** = p<0.001.

Data for Figure S7 B-C): S2_Data.xlsx https://doi.org/10.5281/zenodo.15531855
