# Supplementary material for: A toolkit for mapping cell identities in relation to neighbors reveals conserved patterning of neuromesodermal progenitor populations
Source: PLoS Biol. 2025 Jul 15;23(7):e3003244. doi: 10.1371/journal.pbio.3003244 (PMC12303391; doi:10.1371/journal.pbio.3003244)
Supplement: S8 Fig — (A) Process in identifying and excluding paraxial mesoderm-like clusters in gastruloids. (i) UMAP of TBXT/TBX6 patterning variables; namely normalized fluorescence units (NFI), mean neighbor NFI, local NFI heterogeneity (coefficient of variation). K-means clustering identifies clusters which when (ii) mapped onto the gastruloid localizes with paraxial mesoderm-like clusters. Graphic showing field of view for IF image and overlay of mesoderm-clusters and TF +ve cells. Mesoderm clusters are excluded in downstream analysis. (B) Similar in Fig 3, slingshot pseudotime tools are used in a PCA dimension reduction of smoothed normalized fluorescence units in gastruloids to identify pseudospace. NMP-like cells (NMPLC) are identified in gastruloids using pseudospace gates characterized E8 embryos. (C) Pseudospace values and NMPLC mapped onto the 10um section of a gastruloid using nuclei centroids. NMPLCs can be found not at the tip, but just ‘anterior’ of the tip in the elongation axis. Data for S8 Fig (B–C): Data file 2, https://doi.org/10.5281/zenodo.15802710. (DOCX) [file pbio.3003244.s008.docx]

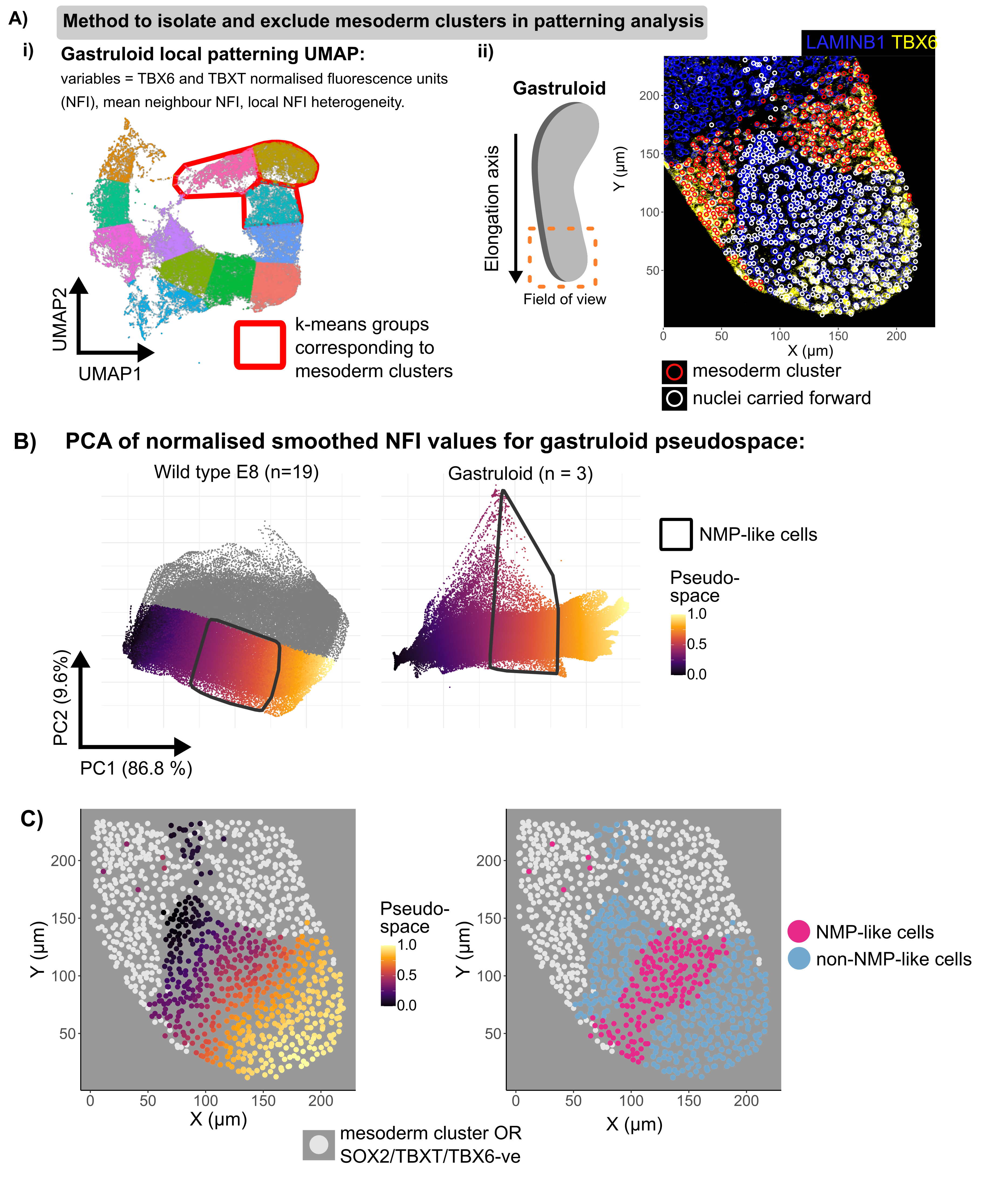


**Fig S8 Methodology to identify NMP-like profiles in gastruloids using pseudospace**

1. Process in identifying and excluding paraxial mesoderm-like clusters in gastruloids. **i)** UMAP of TBXT/TBX6 patterning variables; namely normalised fluorescence units (NFI), mean neighbour NFI, local NFI heterogeneity (coefficient of variation). K-means clustering identifies clusters which when **(ii)** mapped onto the gastruloid localises with paraxial mesoderm-like clusters. Graphic showing field of view for IF image and overlay of mesoderm-clusters and TF +ve cells. Mesoderm clusters are excluded in downstream analysis.
2. Similar in figure 3, slingshot pseudotime tools are used in a PCA dimension reduction of smoothed normalised fluorescence units in gastruloids to identify pseudospace. NMP-like cells (NMPLC) are identified in gastruloids using pseudospace gates characterised E8 embryos*.*
3. Pseudospace values and NMPLC mapped onto the 10um section of a gastruloid using nuclei centroids. NMPLCs can be found not at the tip, but just ‘anterior’ of the tip in the elongation axis.

Data for Figure S8 (B-C): S2_Data.xlsx https://doi.org/10.5281/zenodo.15531855
