# Supplementary material for: A toolkit for mapping cell identities in relation to neighbors reveals conserved patterning of neuromesodermal progenitor populations
Source: PLoS Biol. 2025 Jul 15;23(7):e3003244. doi: 10.1371/journal.pbio.3003244 (PMC12303391; doi:10.1371/journal.pbio.3003244)
Supplement: S9 Fig — 1. Embryo is mounted between coverslips and compressed to partially flatten to expedite image acquisition. 2. Embryos are imaged from both sides to generate two images from different views. 3. Each image is separately processed with single cell segmentation and quantification. 4. The two datasets are registered using the centroids of nuclei. 5. The nuclei with centroids in the half closest to the objective only are selected and carried forward for analysis. (DOCX) [file pbio.3003244.s009.docx]

=


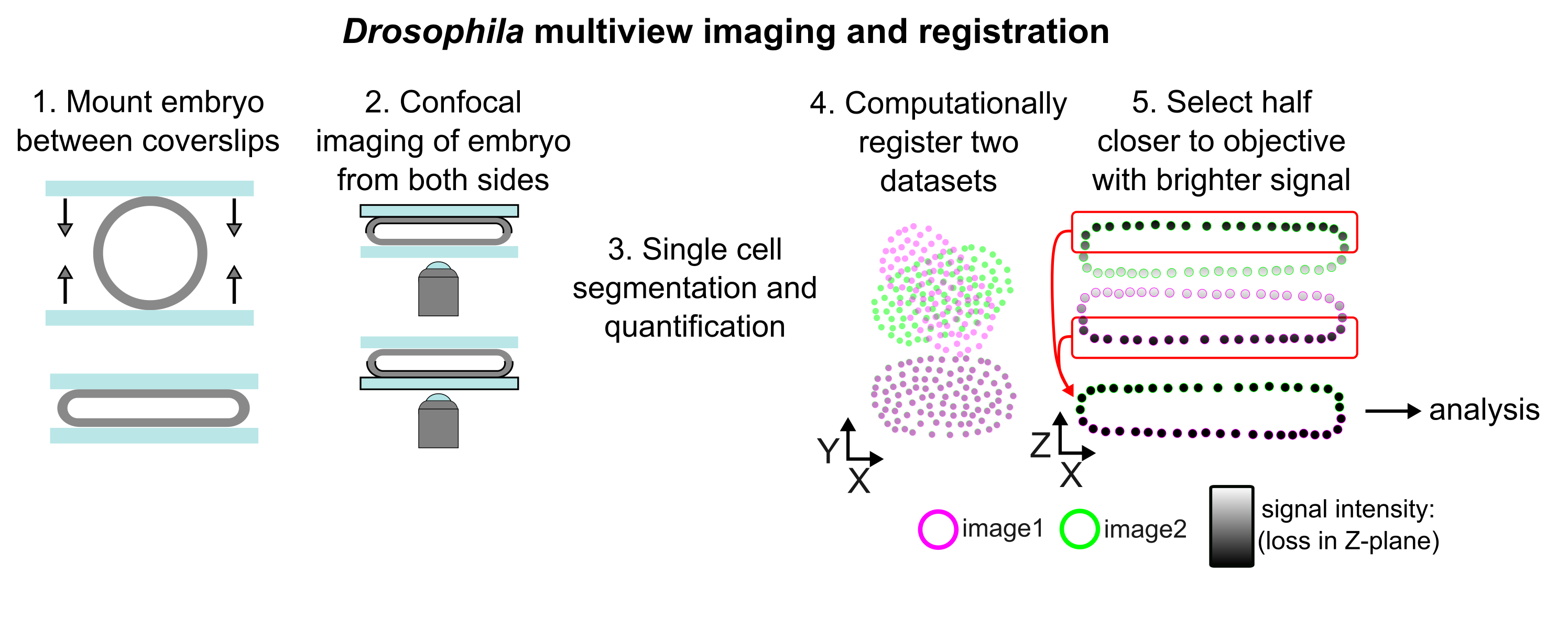


**Fig S9 Steps for imaging and processing Drosophila embryos for PRINGLE.**

1. Embryo is mounted between coverslips and compressed to partially flatten to expedite image acquisition. 2. Embryos are imaged from both sides to generate two images from different views. 3. Each image is separately processed with single cell segmentation and quantification. 4. The two datasets are registered using the centroids of nuclei. 5. The nuclei with centroids in the half closest to the objective only are selected and carried forward for analysis.
