# Supplementary material for: A toolkit for mapping cell identities in relation to neighbors reveals conserved patterning of neuromesodermal progenitor populations
Source: PLoS Biol. 2025 Jul 15;23(7):e3003244. doi: 10.1371/journal.pbio.3003244 (PMC12303391; doi:10.1371/journal.pbio.3003244)
Supplement: S1 Methods — (DOCX) [file pbio.3003244.s010.docx]

**Supplemental methods: details of algorithms**

**Epiblast manifold projection and alignment algorithm steps.**

1. The raw XYZ coordinates of an individual embryo epiblast are manually oriented such that the Anterior Posterior axis aligns with the Y axis, such that the epiblast is as close to symmetric and the Z axis is as flat as possible, creating new XYZ coordinates.

2. Each cell's position along an interpolated principle curve (as measured 0->*n* in um) in the new YZ plane is using found the slingshot package [[45]](https://paperpile.com/c/8qkrSO/nN3g). This metric is used herein as the anterior-posterior (AP) axis position.

3. Starting from 0 in a moving window, cells +/- 20-30um* along from this AP position are isolated. This is the start of the loop; the AP position will increase in steps of ~5um* and run through to the terminal nuclei of the epiblast.

4. In the XZ plane, each cell’s position along the epiblast curve is found using the slingshot package (as measured 0->*n* in um). This is termed the left/right (LR) position value. The direction of position 0->n along the curve is ensured to orient from left to right.

5. The cells expressing the top 10%* levels of T are identified, and the average LR value of these cells is determined as the midline, not any individual cell.

6. The LR midline value is subtracted from all other cell’s LR value, in doing so normalising the position of each cell to the midline. Cells to the left of the midline have negative LR values, and to the right positive LR values.

7. After this normalisation, the distance from the midline as a percentage from the epiblast edge is calculated. First, the LR values for cells to the left of the midline are divided by the LR value furthest cell on the right side (identified by containing a negative LR value), and similarly for the left side (identified by containing a positive LR value).

8. This information is stored and loops back to step 3, where the next AP position is +~5um (a parameter for optimisation) from the previous subset position. The small increase in AP position results in each cell’s LR position being calculated multiple times and a moving window.

9. After the entire AP axis has been run through, the multiple values of LR position and relative LR position for each cell has been calculated, among other metrics. The average of these values from every loop is carried forward.

* = parameters optimised per dataset.

**Epiblast shape registration method algorithm steps**

1. First, the posterior nascent notochord is manually labelled in each epiblast in PickCells, and the equivalent AP position of this is subtracted from each cell’s AP value. Such that position 0 is the start of the notochord.

2. To normalise the length of the primitive streak, each cell's new notochord normalised AP value is divided by the posterior most value, creating a relative position along the primitive streak from the Notochord. This relative AP value is carried forward.

3. Next, the LR position is either maintained as right negative and left positive values are converted to positive values, effectively digitally folding at the midline to combine LR sides of the epiblast.

4. Starting from the posterior end in a moving window, cells from all epiblasts +/- 0.05* relative AP values from the starting position (S) are isolated.

5. The relative LR value is multiplied

6. Then, the process loops back to step 4, where the AP position is increased by a step of 0.015 as a moving window. Again, calculating multiple values of renormalised LR values for each cell. The loop finishes at the posterior most cell’s AP position.

7. After the process has run through, the average of the renormalised LR values for each cell are used as the final normalised LR position.

* = parameters optimised per experiment.

**Neighbour Smoothing and Neural-NMP-Mesoderm pseudo-space.**

TF signal for each nuclei was iteratively smoothed by taking the average TF signal of the nuclei and its neighbours. This new average (AvTF+1) was then used in another round, where the average AvTF signal of a nucleus was calculated (AvTF+2). This was repeated 10 times (AvTF+10) for each TF measured to spatially smooth the TF signal. After which, the log(AvTF+10) signal was used as an input for PCA dimension reduction. The pseudo-space route from Neural to NMP to Mesoderm was identified using the Slingshot package [[45]](https://paperpile.com/c/8qkrSO/nN3g) in PCA1 and PCA2 space. NMP region gates in pseudospace were ascertained by scanning gate values to best fit the bi-fated region in four somite pair embryos, which is then applied to all wild type embryo stages. Gates in pseudospace used to isolate NMP ROIs in ex *vivo* cultured embryos were identified by i) the average pseudospace of node streak border nuclei and ii) the average pseudospace values when mean population TBX6 rises above background along the pseudospace axis.

STREET, K., RISSO, D., FLETCHER, R. B., DAS, D., NGAI, J., YOSEF, N., PURDOM, E. & DUDOIT, S. 2018. Slingshot: cell lineage and pseudotime inference for single-cell transcriptomics. *BMC Genomics,* 19**,** 477.

All numerical data are provided as xl files on github alongside the code used in this study <https://github.com/MattFrenchh/PRINGLE> [https://doi.org/10.5281/zenodo.15531855](https://eur02.safelinks.protection.outlook.com/?url=https%3A%2F%2Fdoi.org%2F10.5281%2Fzenodo.15531855&data=05%7C02%7C%7C11e40a2c861e4c16f88808dd9d8cf882%7C2e9f06b016694589878910a06934dc61%7C0%7C0%7C638839953036342307%7CUnknown%7CTWFpbGZsb3d8eyJFbXB0eU1hcGkiOnRydWUsIlYiOiIwLjAuMDAwMCIsIlAiOiJXaW4zMiIsIkFOIjoiTWFpbCIsIldUIjoyfQ%3D%3D%7C0%7C%7C%7C&sdata=5QHihyejMBQ%2B9TGJJbhSejgma4FfDYQDyjDToMz4zuQ%3D&reserved=0)

**References cited in supplemental information can be found in the reference list of the main manuscript**
