# Supplementary material for: A toolkit for mapping cell identities in relation to neighbors reveals conserved patterning of neuromesodermal progenitor populations
Source: PLoS Biol. 2025 Jul 15;23(7):e3003244. doi: 10.1371/journal.pbio.3003244 (PMC12303391; doi:10.1371/journal.pbio.3003244)
Supplement: Attachment — Submitted filename: 250409 French et al Response to reviewers .docx [file pbio.3003244.s013.docx]

**RESPONSE TO REVIEWERS**

French et al PBIOLOGY-D-24-02571R1

All changes since first submission are highlighted in yellow on our revised manuscript (other than minor typographical corrections).

***Reviewer #1: Identified himself as Jong Kyoung Kim**

Summary: In this manuscript, the authors developed a computational pipeline to quantify and compare cell fate patterning from confocal immunofluorescence imaging data of developing embryos. Based on the tissue landmarks manually provided by the user, cells are positioned using relative coordinates, a key step in ensuring comparisons between embryos. They applied this pipeline to characterize the cell fate transition of neuromesodermal progenitors (NMPs) within the caudal lateral epiblasts of E8.5 embryos, and demonstrated that the patterning of NMPs and the observed heterogeneity of TBX6+ mesodermal derivatives are sensitive to Notch inhibition.

The present manuscript is well written and potentially important in providing a valuable computational pipeline for quantifying and comparing cell fate patterning within developing embryos.

*We thank this reviewer for appreciating the value of our work.*

However, there are several major points that should be addressed to substantiate the authors' claims.

Major points:
1.1.	The authors have nicely demonstrated the utility of their pipeline in the context of cell fate transition of NMPs within the caudal lateral epiblasts. However, as this is an ad hoc approach tailored to the caudal lateral epiblasts (requiring prior knowledge of the corresponding tissue landmarks), it is not clear how this approach is applicable to a more general context of developing embryos. For generalization, the authors should provide a more systematic approach to projecting cells onto relative coordinates based on tissue landmarks, and demonstrate that their approach can be applied to a more general context.

*We have successfully extended our analysis to the following new contexts resulting in two new main figures and one new supplementary figure.*

1) *Chick embryos and cross-species comparisons: new Fig 8 and Supp Fig S9. Chick embryos are over an order of magnitude larger than mouse embryos and have a different architecture, so we used these to exemplify how our pipeline can be further extended to embryos of different shapes and sizes. This facilitates quantitative comparison between two species (mouse and chick), revealing conserved patterning and raising interesting questions about how patterning can so accurately be scaled across the very different sizes of the mouse and chick epiblast.*
2) *Fly embryos: new Fig 9. We present an analysis of drosophila embryos that exemplifies how our pipeline can be applied to a completely different system using a different set of landmarks and coordinates. Notably, fly embryos have an overall 3D elliptical shape but can bend into unpredictable shapes. We were able to demonstrate that our pipeline could cope well with this new set of challenges, making it possible to measure the position of gene expression boundaries across multiple embryos while retaining 3D information. This revealed interesting differences in the scaling of dorsal-ventral patterning between the anterior and posterior ends of the embryo.*

*We are grateful to this reviewer for their suggestion, which now demonstrates that the approach is applicable to embryos of different species, with different shapes and different sizes. This analysis has also broadened the scope of our manuscript by demonstrating the utility of our method for revealing conservation and scaling of patterning across different tissue regions and between different species.*

1.2.	To increase usability and reproducibility, the code for the proposed pipeline should be available, carefully documented and wrapped into a R or python software package.

*We have deposited our code plus explanations onto github (*[*https://github.com/MattFrenchh/PRINGLE*](https://github.com/MattFrenchh/PRINGLE)*) and we reference the code in our revised methods section line 1108. This is also referenced at the end of our supplemental materials.*

1.3.	Figure 3C(ii), D, E: How and why the cells colored by gray were excluded?

*Our aim here was to exclude cells that sit beyond the outer edge of the NMP-containing region. Briefly: we first used our pseudotime analysis to find a spatial trajectory from neural to mesodermal identities. We then took advantage of the fact that neural-mesodermal bipotent regions had previously been established based on functional grafting experiments (Cambray and Wilson 2007; Wymeersch et al. 2016), and set a posterior limit of the ROI based on this information. Some minor manual adjustment was used to ensure a coherent boundary (i.e. to avoid excluding sporadic nuclei that sat within the boundary but would otherwise have been excluded based on gene expression profile). We would like to re-emphasise that our intention here was simply to define the boundary of our region of interest (not to determine the identity of every cell within that region)*

*This information has been added to the methods section p34 and referred within the figure legend.*

1.4.	Figure 3E: How the authors classify the cells into the three regions?

*We have now added this information to the figure legend as follows*

*Legend to Fig 3E: “The boundary between excluded cells (grey) and the broad Neural/Meso progenitor domain (blue) is determined as described in the methods section. Red nuclei correspond to cells with pseudospace values defined in (Cii, D: see also main text) as corresponding to likely NMP identities (‘NMP-like cells’). These map to a U-shaped region that extends beyond the boundaries of the known N-M-bifated regions (black boxes: based on functional grafting experiments* [*(Cambray and Wilson, 2007; Wymeersch et al., 2016)*](https://paperpile.com/c/lcG1hb/ugkb+4HxD)*.” p10*

*This legend refers to the following section of the main text*

*“These values were then mapped back onto the “PRINGLE projection” epiblast (Figure 3D) in order to establish which pseudospace identities correspond to the regions of known NMP potency (black boxes in Fig 3D). On this basis, these values were assumed to represent an NMP-like identity. Nuclei that were defined in this way as having NMP-like identity were then mapped to the epiblast projection” p9*

*And to the following section of the methods section:*

*“To determine which cells should be excluded from our overall region of interest (excluded cells coloured grey in figure 3D, E) we first used our pseudotime analysis to find a spatial trajectory from neural to mesodermal fate. We then took advantage of the fact that bipotent regions had previously been established based on grafting experiments [40,41], and set a posterior limit of the ROI based on this information. Some minor manual adjustment was used to ensure a coherent boundary (i.e. to avoid excluding sporadic nuclei that sat within the boundary)” p34*

1.5.	The CV values are usually inversely proportional to the mean values. Does this relationship affect the interpretation of Figures 5 and 6D?

*We don’t believe this relationship affects the interpretation of Figures 5 and 6D.*

*It is to be expected that variability increases at low expression values due to stochastic effects, and indeed we see some increase in CV at low expression levels for all three markers analysed: TbxT, Sox2, and Tbx6.*

*However, importantly, the CV values for Tbx6 are much higher than those for TbxT and Sox2 at equivalent immunofluorescence intensity values, suggesting that local variability in Tbx6 is not only a consequence of low expression but likely to also be due to some other factor (for example, Notch-mediated lateral inhibition). This is further supported by the non-random distribution of the Tbx6-high cells, and the fact that this distribution is sensitive to pharmacological inhibition of Notch. For these reasons, we suggest that the relationship between CV and mean expression is consistent with our interpretation of the data shown in Figure 5 and 6D.*

*In the manuscript we are careful to avoid making strong claims about the mechanisms that generate local variability. For example, in the discussion section line 807 we acknowledge and discuss the possibility that “local variability between neighbours…. could represent bistability in stochastic cell fate allocation at threshold levels of signalling inputs”. We also state that although we currently favour a lateral-inhibition hypothesis, this would be an interesting area to explore further in future studies, for example using neighbour-labelling technologies.*

Minor comments:
1.6	Figure 1C is not referred to in the manuscript.

*Thank you. We have now included reference to Figure 1C line 145.*

1.7.	"(Blin et al. 2019)" on Line 161-162 should be "[3]".

*Thank you. This has been fixed.*

1.8	An unpaired parenthesis on Line 174.

*Thank you. This has been fixed.*

1.9.	Figure 2B: The tissue landmarks should be clearly marked in a schematic of the epiblast for readers not familiar with embryonic development.

*Thank you for this suggestion. We have added more information to the legend for Fig1A, which shows a schematic of the epiblast in the context of the embryo. We hope this is sufficient to orient the non-specialist.*

***Reviewer #2**

The manuscript entitled "A toolkit for mapping cell identities in relation to neighbours reveals Notch-dependent heterogeneity within neuromesodermal progenitor populations" presents a novel computational toolkit for characterizing cellular identity and patterning during embryonic development. The study focuses on the transition of neuromesodermal progenitors (NMPs) to mesodermal fate in E8.5 mouse embryos. The authors tackle the challenge of analyzing complex 3D tissue structures and cell fate transitions, using computational methods that quantify relationships between cells and their neighbors to understand the spatial patterning of cell identity.

The work makes several key contributions: First, it provides a faithful projection of 3D patterning into 2D that allows for comparisons of different embryos with each other in an unbiased way. Secondly, the authors show by analyzing the relationship between neighbourhood relations and cell identity that SOX2+ and TBXT+ cells tend to be surrounded by cells of similar identity, and TBX6-high cells are surrounded by TBX6-low cells. Finally, they developed a computation method called PRINGLE that is designed specifically to handle the complex curvature of 3D tissues.

Overall, the paper provides valuable insights into how neighboring cell interactions and signalling pathways contribute to the spatial patterning of cell fate decisions during embryonic development. The toolkit it presents will be useful for further studies on cell differentiation and tissue organization in both developmental and disease contexts. The results are scientifically sound and well-presented.

*We thank this reviewer for highlighting the value of our work*

Minor points:
2.1- In Figure 4c, the authors should state how they calculated the confidence intervals.

*We have added this information to the figure legend*

2.2- In Figure 5b, the authors use Tukey's HSD to test whether the coefficient of variation of different transcription factors differs significantly. This test compares pair-wise means between groups but does not directly test for a difference in the coefficient of variation. The authors should use a test that directly addresses the coefficient of variation, such as Feltz and Miller 1996 or Krishnamoorthy and Lee 2014.

*Apologies, the Y axis on our graph was not labelled correctly: we are comparing the mean of many CV values rather than comparing CV values directly. We have corrected this on the revised figure and thank the reviewer for spotting this error.*

*We have taken this opportunity to reexamine the choice of statistical analysis: as explained above, we are not directly comparing individual CV values of a single sample population, we are comparing multiple replicates and groups represented by a mean of multiple CVs. We performed extra testing and simulations to confirm the assumptions of normality and equal variance required for Tukey’s HSD. However, we realise that our data should be treated as a paired test, and now present analysis using a paired t-test and Benjamini Hochberg post-hoc correction in Figure 5b.*

2.3- In Figure 5f, the end points of each line have larger confidence intervals compared to the other data points. This suggests that the authors used a binning of pseudo-space to create this plot. If this is correct, they should state this in the caption including the bin size. The author should also indicate how they calculated the confidence intervals and in which form a potential binning of the data was considered for this.

*Cells were binned into even intervals and only cells within these intervals were considered for confidence intervals and statistical summary metrics. No sliding windows or local smoothing filters were implemented here. This information has been added to all relevant figure legends.*

2.4- In the method section entitled "Nuclear intensities smoothing" the authors state that they run a PCA on the variable $\log{(AvTF+10)}$. They do not state whether they centered or scaled the data for this purpose. Does scaling or centering make a difference in the results?

*We normalise the smoothed values using the same values used to normalise the raw values. We have explained this in our revised text line 1044.*

***Reviewer #3:**

Pattern emergence and transformation is one of the key features encountered when studying developmental processes. Yet very few tools or analytic pipelines exist that allow for a quantitative approach to assess and compare features of the local microenvironment such as the expression of markers in the neighbouring cells and local coherence vs heterogeneity in patterning of cell states.
The work of Lowell and colleagues aims to bridge this gap as they describe a new pipeline that allows the comparison of patterns across multiple individual embryos/organoids or chosen ROIs in such structures. The authors used existing E 8.5 mouse embryo landmarks to compare emerging neighbourhoods' patterns between different embryos and subsequently used this pipeline to demonstrate that TBX6 positive cells in the NMP progenitor region tend to be surrounded by TBX6-negative cells and organised in a non-random distribution. By comparing patterns of the wt embryos with embryos that had been subjected to Notch inhibition, the authors found that discovered local heterogeneity of TBX6 depends on Notch signalling.

Importantly, the pipeline the authors proposed seems to be suitable to make in vivo/in vitro pattern comparisons, which in the opinion of this reviewer makes it an extremely valuable tool for any scientist interested in pattern formation and modelling of biological systems.

Before this elegant work is considered for publication, there are several points that need attention.

*We thank this reviewer for their supportive comments*

Major points

3.1 MM section - I am assuming that mice were naturally mated? Also, were all embryos collected from the same litter or several litters (I am assuming the latter is true but how many litters were analysed?) As the different shapes of the embryos were mentioned in the manuscript, have the authors checked how litter to litter variability influences the outcome of the PRINGLE method (and subsequent expression/gradient/neighbourhood etc analysis)? How similar were the results from the same stage embryos from different litters?

*The mice were naturally mated and collected from three different litters. The demarcation of the NMP region (white lines shown in Figs 4 & 5) indicates variability in the NMP region between all embryos examined, which serves as a useful indicator for differences in the outcome of the PRINGLE method and subsequent expression analysis. As is apparent from these figures, we see very little variability in the location of the putative NMP region across all embryos analysed, regardless of litter-of-origin. This is perhaps unsurprising given that we were able to stage each embryo precisely by somite number to avoid variability due to differences in developmental timing between individuals.*

3.2 I am also unsure how simply using marker expression for the PRINGLE method to analyse gastruloids (data from fig 6) can work. As far as I can say, gastruloids often vary in shape, size and also expression pattern domains. How did the authors choose which expression patterns were the most representative/similar to the embryos? Alternatively, if the authors created an "average" projection using several gastruloids different in size, shape and/or expression pattern, how confident can we be that the final projection was representative/correct?

*We agree this was not sufficiently clear in our manuscript. We don’t perform an averaged projection for the gastruloid analysis. Rather, our aims here were as follows:*

*a) We aimed to map the NMP region in a relatively unbiased way, avoiding the usual approach of setting arbitrary thresholds for fluorescent intensity. We did this taking advantage of the fact that we can confidently identify an NMP immunofluorescence signature in embryos based on data from grafting experiments* [*(Cambray and Wilson, 2007; Wymeersch et al., 2016)*](https://paperpile.com/c/lcG1hb/ugkb+4HxD)*, which is consistent with the protein signature that we examine in more depth in the current work. We can then identify cells with a similar marker-expression-signature within gastruloids and use this information to map a putative NMP region.*

*b) We use neighbour-analysis to measure local variability in and around the NMP region. We use these data to measure the overall degree of heterogeneity across a particular region, but not to map the averaged spatial distribution of CV values.*

*We have modified the text to clarify this point as follow: “This analysis differs from that shown in Figure 5 because our aim here was to measure overall levels of local variability in relation to cell identity, not to map CV values onto an averaged projection.” Our results section also includes an explanation of why the analysis of gastruloids is more limited than that of embryos because “a detailed like-for-like comparison would require knowledge of the fates of cell neighbourhoods that is currently not available in cell culture model systems” line 490.*

*Please note that although our gastruloid data is more limited than our mouse embryo data, we have now strengthened the case for generalisability by analysing two additional embryo contexts: fly embryos and chick embryos new Figs 8 and 9: see also point 1.1 in our response to reviewer one above*

3.3 … Have the authors repeated the experiments several times and tested how similar are the projections from different experiments? Will these results be comparable between different labs (considering that efficiency of the whole process varies between labs and may influence expression domains?)

*The gastruloid data represents data from three experiments with three gastruloids per experiment. We have included this information in the figure legend.*

*The reviewer makes a good point: gastruloid formation can vary somewhat between labs. Importantly, we are not making strong claims about the biological significance of our gastruloid observations: our main conclusion is that “our analysis methods could be useful for comparing local variability between different experimental models”. Our pipeline could perhaps, in future, prove useful for examining reproducibility of gastruloid patterning phenotypes between labs.*

3.4 Although, I can clearly see the advantage of using the pipeline presented by the authors
I am not sure if gating the populations manually for positive/negative classification of the nuclei is the best strategy here. The authors made such a commendable effort to standardise most of the whole analysis and this specific step, in my opinion, introduces subjectivity to the whole process.

*Our view is that even ‘automated’ methods for gating usually depend on some balance between user-decisions and objective information. An important aspect of our approach for identifying NMP populations is that it incorporates objective information from fate maps rather than relying entirely on subjective arbitrary thresholds. In the case of gastruloids, this is not possible, and we note this in the text as a limitation line 491.*

Minor points

3.5 There is an inconsistency of referencing through the whole text. The authors should decide on the referencing method and make it consistent in the whole manuscript.

*Thank you. We have now fixed this.*

3.6 It would be good to add E8.5 mouse embryos as nowhere in the title or abstract it is clear what systems the authors refer to.

*The revised abstract now specifies that we study E8.5 mouse embryos. We’ve also mentioned within the abstract that the approach has been extended to examine patterning of chick and fly embryos.*

3.7 Fig 2C - I am not sure how TBX6 staining on this panel corresponds to the staining presented in fig 1B (SOX2 and TBX staining look similar in both figures).

*We agree with the reviewer that the staining pattern in Fig 1B is difficult to interpret! This is an example of the type of problem that motivated our development of the PRINGLE method. To explain:*

*Figure 1B shows a conventional confocal image. Due to the curved shape of the embryo, an optical section of this type gives a misleading impression of the overall patterns of gene expression. As noted by the reviewer, this causes particular confusion in the case of TBX6 because TBX6 marks a layer of mesoderm that sits beneath the epiblast layer, but curves around to sit within the same optical plane. We have clarified this point in the revised text line 145.*

*This problem is one of the reasons we developed the “PRINGLE” method, which aims to capture patterning across the curved 3D epiblast in a 2D projection (Fig 2), while excluding the presomitic mesoderm layer from the analysis.*

3.8 It would be good to explain what the difference between black and red arrows in Fig S4 3D is.

*Black arrows indicate positive values while red arrows indicate negative values. We have added this information to the figure legend for Fig S4: thank you for spotting this omission.*

3.9 Fig 3 description is not finished (see line 246)

*Thank you! We have corrected this.*

3.10 Fig 5D description, I am unsure what "solid lines" on the graph the authors refer to.

*Apologies, this was an error. It has now been corrected.*

3.11 Fig 6 A description, What fig the authors had in mind by saying "(further characterisation fig SX)"?

*Thank you for spotting this. We have now corrected this to say “Fig S7”.*

3.12 I found the section titled "Local heterogeneity in TBX6 is sensitive to Notch inhibition" having not enough references to the fig 7, especially in the last 3 paragraphs. Adding appropriate references to the figure per each main finding, would help the reader to follow this section better.

*Thank you for spotting this omission. We have now added references to the figure for each main finding.*

3.13 In Fig 7F description, the authors wrote: "Note the statistically significant decrease in the CLE and NSB like regions for both TBXT and TBX6" but I cannot see TBXT data on that figure.

*Apologies, this was an error. We have corrected this by deleting references to TBXT.*

***Reviewer #4:**

Summary
The manuscript Matthew French et al. presents computational methods for quantitative analysis of cell patterning in complex 4D datasets. To this end, the authors focus on the axial progenitor region in mouse, containing neuromesodermal progenitors (NMP) expressing variable levels of different transcription factors across space and time. They combine previously developed and new computational pipelines to: 1) simplify visualization of 3D patterning in 2D, 2) define spatial domains from multiple averaged samples, 3) use cell-neighbor relationships to define marker expression changes and mesoscale patterning, and 4) apply this to various experimental conditions. One of the relevant findings, facilitated by their cell-neighbor analysis, is that TBX6 expressing cells in the NMP domain have a non-random distribution. They suggest a potential lateral inhibition mechanism for mesoderm commitment in this region.
I particularly value the development of pipelines to analyze and visualize 3D tissue patterning across averaged samples and experimental conditions. This work is of significant value for the field and will facilitate the study of tissue patterning from developmental biology to disease modeling. However, I have major questions and concerns, related with the explanation of the methods, interpretation of some results and the comparison of this method with existing ones, that will need to be addressed prior to publication. My specific comments are included below.

*We thank the reviewer for their appreciation of our work.*

Major comments

.	A major concern is that the methods presented here are not accessible to non-experts and cannot be reproduced with the information currently provided. These are my suggestions:

4.1	Provide all the code through an Open Access platform. Reference the algorithms presented in Supplementary Material to the corresponding code.

*We have deposited our code plus explanations onto github (*[*https://github.com/MattFrenchh/PRINGLE*](https://github.com/MattFrenchh/PRINGLE)*) and we reference the code in our revised methods section line 1108. This is also referenced at the end of the supplementary methods.*

4.2	Reduce the use of jargon and explain why a particular method was used to non-experts. For example, in line 361: "We then computed the earth move distance between the observed…" the concept was not explained before and there is no further information in supplementary information.

*We agree that this term will be familiar to many biologists. We have provided a reference in the main text line 393, and in the methods section we provide an explanation of the term and explain how the metric was calculated line 1085.*

An important goal of this manuscript is to avoid arbitrary selection of regions based on marker thresholding. However, there are multiple examples of manual selection of regions or cell selection:

4.3	Line 166: "The epiblast is manually isolated using PickCells software". Please show how you define the region of interest in different embryos.

*This was based on morphology: the epithelial cells are clearly different in shape from the underlying PSM or the notochord. Our revised manuscript includes a new automated approach for defining the epiblast based on machine learning, avoiding the need to manually demarcate the tissue using visual inspection of cell morphology in situations where this is not straightforward (new Fig 8).*

4.4	Line 174: "…node (manually labelled)…". While the authors provided one example in Figure S2, it would be best to show how they select the same region across embryos to avoid introducing bias.

*The node is a visually distinct landmark defining the anterior end of the primitive streak, so we feel it is straightforward to identify this region in an unbiased manner. If the reviewer feels it is important to provide additional images then we will be happy to do that.*

4.5	Line 705: " Ten of the 19 segmented embryos were manually edited in PickCells to correct for segmentation errors." Please explain why only ten were manually edited.

*Manual correction is a labour-intensive process (3-4 days per embryo). After manually editing the first ten embryos, we used these as a ground-truth to establish whether manual editing actually made a difference to the final analysis. We established that it did not, and so we proceeded to analyse the remaining 9 embryos without manual correction. We have added this information to our revised methods section line 1021.*

4.6	Line 722: " Nuclei were classified as positive or negative by gating the populations manually after combining all the replicates". Please explain why this needs to be done manually or provide alternatives to classify the cells automatically.

*Our view is that even ‘automated’ methods for gating usually depend on some balance between user-decisions and objective information. An important aspect of our approach for identifying NMP populations is that it incorporates objective information from fate maps rather than relying entirely on subjective arbitrary thresholds. In some cases, for example for our gastruloid data,, this is not possible, and we note this in the text as a limitation line 490.*

4.7.	Previous computational methods were developed for analysis of 3D and 4D datasets across samples (References 18-31). One example is MorphoGraphX 2.0 (Ref 27) which includes a software interface, while others have custom pipelines to analyze patterning across averaged samples (Ref 31). To demonstrate the significance of the new method in this manuscript, it would be important to compare their analysis with at least one of these existing methods. If none of the previous methods allows for this type of analysis, please discuss why.

*We agree that there are other excellent 3D image analysis softwares as referenced in our manuscript, but we believe none of these offer an out of the box solution for the particular sets of challenges that we tackle in this paper.*

*MorphoGraphX 2.0 is a powerful tool and we are aware that this software allows for example to graphically define cells as landmarks and then compute geodesic distance in 3D tissues. However, defining the midline manually in a thick multilayered structure such as the epiblast is challenging and error prone and we think that using a moving window of signal intensity to automatically find the midline, as we do in our method, is better suited in this case.*

*The excellent approach described in McDole et al projects the entire embryo using a mercator projection and broadly assumes a spherical coordinate system: this works well for studying AP patterning, particularly in earlier mammalian embryos, before the embryo undergoes turning and elongation, whereas our method is well suited for larger and longer embryos, and facilitates analysis of LR patterning.*

*We believe we would not have been able to generate the data we present in this paper using any one single existing method (Currently, MorphographX, does not provide functionality to register and project multiple samples into one visualisation and TGMM has no function to find landmarks and compute geodesic distance). Our approach complements these other methods, rather than claiming to be superior to them.*

*We appreciate that these tools possess a plugin mechanism allowing third parties to extend the current functionalities of these softwares. We decided to build our toolkit using python and libraries that are widely known to the community so that our toolkit can be used independently and later on ported as a plugin to any of these tools.*

4.8.	The use of pseudotime tools to represent pseudospace is an interesting approach (Figure 3). However, the conclusion that NMP region extends beyond the known fate maps needs clarification. The authors should explain how they allocate fate map regions (black boxes) from previous studies to their own epiblast projections. Further, they should explain how they define a U-shaped region from pseudospace and those allocated bi-fated regions - are there thresholds? They did not explain any of this in the text or Methods.

*Thank you for suggesting this, we agree further explanation was needed*

*This has been explained in the methods p34, and summarised below:*

*Defining a putative NMP region: We first allocate NMP-fated regions (black boxes) based on fate-mapping experiments that use microdissection and grafting of these regions to determine their fate* [*(Cambray and Wilson, 2007; Wymeersch et al., 2016)*](https://paperpile.com/c/lcG1hb/ugkb+4HxD)*. We allocated the size and position of these regions using exactly the same approach as was originally used to identify regions for microdissection and grafting* [*(Cambray and Wilson, 2007; Wymeersch et al., 2016)*](https://paperpile.com/c/lcG1hb/ugkb+4HxD)*: the width of the regions is set to match the width of the node, and the position of the boxes is determined in relation to the position of the node.*

*To define the U shaped putative NMP-like region, we identified pseudospace values corresponding to the known NMP-fated regions (i.e. for cells within the black boxes) and then searched for other regions with the same pseudospace identity: these other regions, together with the boxed regions, form the U shape corresponding to a region of NMP-like pseudospace identity.*

4.9.	The number of samples analyzed for some developmental stages should be increased to extract representative conclusions. Specifically, there were only 2 samples analyzed at somite pair stage 10-12.

*We agree that data from two embryos is not sufficient to draw conclusions. The analysis of this developmental stage (SP10-12) does not contribute to the overall conclusions of our paper, and could remove these data if the reviewer and editor so wish.*

*However, our preference would be to keep these data in the paper, given that the data are clearly labelled as n=2 and that they form part of a continuum of stages that are closely spaced in time. These tentative observations indicate that patterning may be broadly maintained over this continuum of developmental stages, and they could be of interest to specialists in the field even though they are not important for the overall findings of our paper.*

4.10.	I have major concerns related to the Notch inhibition experiments:

*These concerns of the reviewer are listed below as points 4.11-4.14 along with our responses.*

4.11	The authors claim that Notch manipulation does not affect shape and size of the cultured epiblast (Line 486). However, Figure 7B clearly shows size differences between control and treated conditions. If this is true, an explanation about how to apply their projections and analysis methods to compare both conditions is needed.

*We have considered whether any effects of the Notch inhibitor on shape and size might invalidate our analysis. There is also variation in shape and size within the control population (which, as we state earlier in the manuscript, was one of our motivations for developing the PRINGLE normalisation method). While there may be additional differences in shape and size between Notch-inhibited and control-treated embryos, these were not extreme and did not reach statistical significance in our sample size.*

*We state in the text that Notch manipulation does not grossly affect shape and size, but we did not mean to exclude moderate effects. We have now adjusted the text to make this clearer line 530. The text now reads “ indicating that the overall shape and size of the epiblast region was not grossly affected by Notch inhibition (although we do not exclude the possibility of moderate effects on shape and size)“*

*Given that the PRINGLE method is designed to normalise analysis to overall size and shape, we feel our approach is reasonable. However, we do appreciate that our Notch experiments are relatively preliminary, and serve to illustrate the utility of our method rather than to draw strong conclusions about the role of Notch. We make it clear in our discussion (p 806 onwards) that further work is required in order to exclude alternative explanations for TBX6 patterning. We have also now removed reference to Notch in the title of our paper (see point 4.14 below)*

4.12	The authors claim that there is a bilateral symmetry of the embryo in this region and they combine both sides into single projections (Line 489 and Figure 7D-F). This is not consistent with their previous demonstration of left-right asymmetry (Figure S3, Line 791). They should repeat the analysis separating the left and right sides.

*This is an important point that we considered carefully when deciding how to display our data. We did originally display bilateral patterning (left & right sides not combined) but it became difficult to fit all such images into Figure 7 in a format that allowed for clear comparison between control and Notch inhibited embryos.*

*We then examined data displayed as “half-side” visualisations. Importantly, this shows the averaged pattern combined from both sides of multiple embryos rather than simply ignoring one side of each embryo.*

*We found that the patterning was sufficiently symmetrical that this ‘half-side’ approach didn’t make any difference to the overall conclusions when compared to the original bilateral mapping that showed both left and right sides. It also made the data much easier to visualise and interpret, particularly when comparing Notch inhibited and DMSO-treated embryos. We hope this explains our decision to display the data in this way.*

*Please note that while considering the reviewers comments we took the opportunity to redesign Fig7 to improve clarity. For example, the X and Y axes are now scaled in relative units to more accurately represent the epiblast shape, and the colour palette has been adjusted to improve contrast.*

4.13	The relationship between Notch and TBXT6 has been previously demonstrated in different contexts. The authors should cite and discuss prior knowledge and how this relates to their findings.

*Thank you. We have included this information and added citations to support this point line 521.*

4.14	Based on this and the fact that their observations are still quite preliminary and do not allow to conclude whether Notch signaling is the source of local heterogeneity in the NMP, I think the Title should not include Notch.

*We agree. We have removed reference to Notch in our title.*

Minor comments

Introduction:
4.15.	The authors should try to improve the citations of previous related literature. For example, in Lines 59-63 they cite some methods for 3D data analysis across samples but there are others

*Thank you. We have added the reference Blanc et al suggested by this reviewer line 75.*

4.16	Line 67: "…known regulators of cell lineage, and has a well-defined fate map". A reference is missing here.

*Thank you. We have added three references to support this point line 84.*

Results and Figures:
4.17.	Line 118: "It is not easy to determine by eye whether these sporadic TBX6+ cells are restricted to a particular location or expressed in a particular pattern". There are previous methods that allow to not do this by eye. Please comment on why those previous quantitative computational approaches were not sufficient for this.

*We are unsure about the methods that this reviewer is referring to but we agree that there are image analysis tools that can be used to quantify the level of fluorescence in individual cells in 3D (Cell Profiler for example). To investigate patterning, the standard approach in the field is to define distinct categories of cells using a gating strategy on fluorescence values, either manually or with clustering methods. Further analysis is then carried out using discrete cell categories. While useful, this approach overlooks biologically meaningful graded changes in identity which occur over complex 4D trajectories. Here, we quantify patterning directly, using continuous fluorescence levels within each cell in relation to the continuous levels within neighbours and devised a strategy to compare these patterns between embryos. We are not aware of any currently available method allowing us to perform these analyses out of the box.*

4.18.	Line 162 (Blin et al.) should be a Reference number instead.

*Fixed. Thank you.*

4.19	Lines 209-211: on the comment"…this marker-based approach presents challenges because both TBXT and SOX2 are expressed in graded distributions, meaning that gating for positive cells can be somewhat arbitrary." There are methods allowing non arbitrary selection of thresholds, such as 'Mixture analysis' (Reference 9). Please discuss why this is not enough and the authors still use manual gating for cell selection.

*Our view is that even ‘automated’ methods for gating usually depend on some balance between user-decisions and objective information. An important aspect of our approach for identifying NMP populations is that it incorporates objective information from fate maps rather than relying entirely on subjective arbitrary thresholds. In some cases, for example for our gastruloid data,, this is not possible, and we note this in the text as a limitation line 490.*

4.20	Figure 3 Legend. Add reference in (B). Clarify how you obtained (E) from (D).

*We have added the missing reference and clarified how we obtained E from D (Fig 3 legend, p10).*

4.21.	Line 265. Please add a reference with specific examples.

*We have added two references to reviews that include a number of specific examples line 290.*

4.22	Figure 4C: please improve visualization of the curves with respect to the shading area. Potentially change the background color for the plots to help visualize this.

*Thank you for this suggestion, we have modified these plots*

4.23	Line 332: please provide a more specific example Reference.

*We have added a reference that explains the concept of lateral inhibition line 360.*

4.24	Line 344: here the analysis considers cell-neighbors heterogeneity. Please explain how this can be averaged between different samples where cell resolution is lost.

*The information we present on the averaged image represents the distribution of CV values, i.e. a map of where cells tend to be different from their neighbours (high CV) and where they tend to be similar to their neighbours (low CV). We agree this was not sufficiently clear: we have added a sentence to explain this (line 374 onwards).*

4.25	Figure 5F: please change the colors between Simulated and Observed so that they can be distinguished better.

*Thank you for this suggestion, we have modified these plots.*

4.26	Line 364: the authors claim that TBXT local heterogeneity corresponds to a random distribution. However, the data in figure 5G (ii) shows a significant difference in distribution for TBXT in the transition zone between NM and Mesoderm. Please explain.

*We agree that the TBXT distribution may not be random: we have removed this claim from our manuscript (it was not relevant to our main conclusion here, which is that TBX6 local heterogeneity appears to be non-random).*

4.27	Line 411: the authors use hES derived monolayers, and they should explain why they chose a different species instead of mouse. Could this be another source of variability?

*Yes, this is a good point- the difference in species could be an additional source in variability (although this is very unlikely to be the only reason that differentiation doesn’t display coherent embryo-like patterning in unconstrained 2D cultures).*

*We chose to examine hES cells as an example of how we could use our analysis pipeline to quantify variability in a completely different system. We selected hESC for this mainly because the NMP differentiation protocol has been optimised for human pluripotent cells.*

*We did not set out to examine the various different factors that could contribute to variability in this context: this could include species-specific effects but is likely to include several other contributory factors.*

4.28	Figure 6C: SOX2 also presents higher variability in the density than the other factors. The authors should comment on that result.

*This likely relates to the fact that the populations being examined in the embryo include a higher proportion of Sox2+ cells compared with the populations being examined in gastruloids and hNMPs. This is one example of a limitation in comparing embryos with cell-culture systems due to difficulties in focusing on exactly equivalent populations. This broader point is already discussed within our results section as a limitation to the analysis. We have not expanded on this further in our revised manuscript given that our manuscript is already rather long.*

4.29	Line 499: substitute "T" by "TBXT".

*Thank you. This error has now been corrected.*

4.30	Figure 7F: adding a Supplementary Figure showing some examples of the original data used for this figure showing TBX6 distribution changes between controls and treated samples would be helpful to correlate this 100-cell grid patterns to the original data.

*Does this refer to changes in the expression pattern of TBX6? The data shown in Figure 7D shows the TBX6 distribution change between controls and treated samples.*

4.31	Figure S6 (b). Please use colors that can help distinguish between Observed and Synthetic curves.

*Thank you for this suggestion, we have modified these plots.*

Discussion:

4.32.	Line 546: the statement "comparison between samples at the single cell level" is not accurate given the averaging and comparisons are not done at that resolution.

*Our initial analysis is performed at the single cell level, but these single-cell data are then further processed in order to summarise behaviors of cells, perform statistical analysis, and map trends in patterning. We therefore think it is reasonable to retain this statement.*

4.33	Please discuss how this method overcomes previous methods challenges (see Major comments).

*We feel that the first section of our results lays out the challenges that our method is designed to overcome: we did originally include similar points in our discussion but this felt repetitive and so we removed this section of the discussion in an attempt to keep our manuscript to a reasonable length.*

*Please see response to point 4.7 above, where we explain that we are not aiming to improve directly on the functionality of any other single software package, but rather to generate a novel pipeline to overcome a set of problems that can’t be directly addressed in the same way by existing packages (to our knowledge).*

4.44	Please comment on potential limitations of your study and suggest ways to improve it in the future.

*We have added discussion of potential limitations of our imaging pipeline and opportunities for improvement (line 826 onwards). An earlier section of the discussion also outlines some limitations to our conclusions about Notch as a regulator of NMP patterning, with suggestions for future work (line 806 onwards).*

Methods:

4.45	Line 661: is 36C incubation needed? (37?).

*Apologies, this was a typo, now corrected*

4.46.	Line 665: what is DAPT? Please provide references for reagents.

*We have added an explanation and a reference (line 906).*

4.47	Line 683-686: please provide the concentration used for the different antibodies.
*We have added this information (line 937).*

4.48.	Line 708: please specify the confocal microscopy platform used.

*This was a Leica Sp8 confocal microscope . This has been added to the text (line 994).*

_______________________________________________

**References cited in the Response to Reviewers**

[**Cambray, N. and Wilson, V.** (2007). Two distinct sources for a population of maturing axial progenitors. *Development* **134**, 2829–2840.](http://paperpile.com/b/lcG1hb/ugkb)

[**Farkas, D. R. and Chapman, D. L. eds.** Regulation of Tbx6 expression by Notch signaling Phillip H.](http://paperpile.com/b/lcG1hb/bNP4)

[**Wymeersch, F. J., Huang, Y., Blin, G., Cambray, N., Wilkie, R., Wong, F. C. K. and Wilson, V.** (2016). Position-dependent plasticity of distinct progenitor types in the primitive streak. *Elife* **5**, e10042.](http://paperpile.com/b/lcG1hb/4HxD)

[**Yasuhiko, Y., Haraguchi, S., Kitajima, S., Takahashi, Y., Kanno, J. and Saga, Y.** (2006). Tbx6-mediated Notch signaling controls somite-specific Mesp2 expression. *Proc. Natl. Acad. Sci. U. S. A.* **103**, 3651–3656.](http://paperpile.com/b/lcG1hb/QF2T)
