# Supplementary material for: A toolkit for mapping cell identities in relation to neighbors reveals conserved patterning of neuromesodermal progenitor populations
Source: PLoS Biol. 2025 Jul 15;23(7):e3003244. doi: 10.1371/journal.pbio.3003244 (PMC12303391; doi:10.1371/journal.pbio.3003244)
Supplement: Attachment — Submitted filename: 2500528 French et al Response to reviewers .docx [file pbio.3003244.s014.docx]

**RESPONSE TO REVIEWERS**

French et al PBIOLOGY-D-24-02571R2

Reviewer #1, Jong Kyoung Kim (note, reviewer 1 has signed this review): The authors have satisfactorily addressed my comments.

Reviewer #3: The authors answered satisfactory all my main points. Providing additional analysis of chick and Drosophila embryos significantly improved the manuscript and demonstrated that the pipeline described in the manuscript can be employed to many diverse systems. 
I have only one minor comment. Figure 8 legend needs to be revised. By the look of it, panel F and G description was swapped/mixed up


Reviewer #4: I have now read the Revised manuscript, and I feel it is now acceptable for publication, as the authors have clearly addressed all my concerns and made important changes to the manuscript.

***Author response:***

*We are glad that all three reviewers agree that we have addressed their concerns.*

*We have corrected the error in Fig 8 legend as suggested by Reviewer #3.*
